# ﻿*Schiedeawaiahuluensis* (Caryophyllaceae), an enigmatic new species from Kaua'i, Hawaiian Islands and the first species discovered by a drone collection system

**DOI:** 10.3897/phytokeys.247.130241

**Published:** 2024-10-09

**Authors:** Warren L. Wagner, Stephen G. Weller, Ann K. Sakai, Ben Nyberg, Kenneth R. Wood

**Affiliations:** 1 Department of Botany, MRC-166, National Museum of Natural History, Smithsonian Institution, P.O. Box 37012, Washington, DC 20013-7012, USA National Museum of Natural History, Smithsonian Institution Washington United States of America; 2 Department of Ecology and Evolutionary Biology, University of California, Irvine, CA 92697, USA University of California Irvine United States of America; 3 National Tropical Botanical Garden, 3530 Papalina Road, Kalaheo, HI 96741, USA National Tropical Botanical Garden Kalaheo United States of America; 4 University of Copenhagen, Natural History Museum of Denmark, Copenhagen, Denmark University of Copenhagen Copenhagen Denmark

**Keywords:** Caryophyllaceae, conservation, drone exploration, Hawaiian Islands, Kaua'i, *Schiedea*

## Abstract

During a survey by the National Tropical Botanical Garden drone team, an enigmatic *Schiedea* was observed in December 2021on steep, rocky cliff faces of the Waiahulu Valley in the Waimea Canyon of Kaua'i. Subsequently, another survey was conducted in March 2022 and, by use of a remotely controlled cutting device suspended below the drone, the first herbarium specimen was collected, as well as a seed collection of an undescribed cliff-dwelling species of *Schiedea*. Detailed study of the collections and plants grown at the University of California, Irvine greenhouse showed that it had enlarged, somewhat whitish sepals similar to those of cliff-dwelling *S.attenuata* (the sole species in sect. Leucocalyx), yet differed significantly from all other species in the genus. It also shares with *S.attenuata* a woody habit, hermaphroditic flowers, coloured nectar and styles 5 to 7 or 8. We describe it here as *S.waiahuluensis* given the only known localities are on the cliffs of this valley and place it in an enlarged sect. Leucocalyx. With the discovery of this new species, there are 36 species in this Hawaiian endemic genus.

## ﻿Introduction

Drone technology is advancing quickly and has become an effective tool for botanical surveys of cliff environments (Nyberg et al. 2023). Drone imagery with very high resolution has greatly increased our understanding of the distribution and abundance of many rare plant taxa. In Hawaii, researchers have been developing drone-based sampling systems capable of collecting plant material remotely. These platforms are being used to reach inaccessible areas to monitor the spread of forest pathogens (Perroy et al. 2022) and to conserve critically endangered plants (La Vigne et al. 2022).

While monitoring the walls of Waimea Canyon, Kaua'i for rare plant taxa and using a drone to photograph selected study sites, staff at the National Tropical Botanical Garden (NTBG) documented an enigmatic *Schiedea* in December 2021. On first examination of our images, we speculated that the unique shrub could possibly represent the extinct Kaua'i species, *Schiedeaamplexicaulis* H.Mann, last observed in 1855, or perhaps a new species. Subsequently, we returned in March 2022 and used a remotely controlled cutting device suspended below the drone to make the first herbarium specimen and seed collections of the *Schiedea*. On close examination of our herbarium specimen, it became immediately clear that we had discovered a new *Schiedea* species, bringing the total number of this Hawaiian endemic genus to 36 species (Wagner et al. 2022), with twelve species occurring on Kaua'i as single-island endemic taxa (Wagner et al. 2005). This discovery is likely the first time an undescribed species has been located and collected via drone, demonstrating the profound significance of unmanned aircraft systems in the conservation and prevention of plant extinctions.

## ﻿Methods

For the survey portion of this work, we deployed a DJI Phantom 4 pro quadcopter drone. This is a consumer-grade platform that was chosen for its portability and high-resolution image sensor (20 megapixels). Still photographic images were collected approximately 5 m from the cliff surface to allow adequate resolution for the location and identification of small plants.

Photographs taken by the drone were reviewed and classified by species in Adobe Lightroom software. Image classification is a manual process that relies on visual acuity and expert field knowledge. The high-resolution imagery is examined for species identification and phenology, to track plant distribution and abundance and also to guide field operations. GPS location information is embedded into each image file which assists in locating access points and re-finding specific individual plants.

Once the collection target was selected, we deployed an Outreach Robotics Mamba, suspended under a DJI Matrice 300 drone. The Mamba is a robotic sampling manipulator designed to grab, cut and collect plant material in vertical cliff habitats using propellers to advance the device towards an adjacent cliff in a swinging motion, while keeping the carrying drone clear of obstacles.

## ﻿Taxonomic treatment

### ﻿ Schiedea waiahuluensis

Taxon classificationPlantaeCaryophyllalesCaryophyllaceae

W.L.Wagner, Weller, B.Nyberg, & A.K.Sakai sp. nov.

6FAD561E-F979-5C33-A3BD-B0BE42709D57

urn:lsid:ipni.org:names:77349836-1

**Figure 1.**
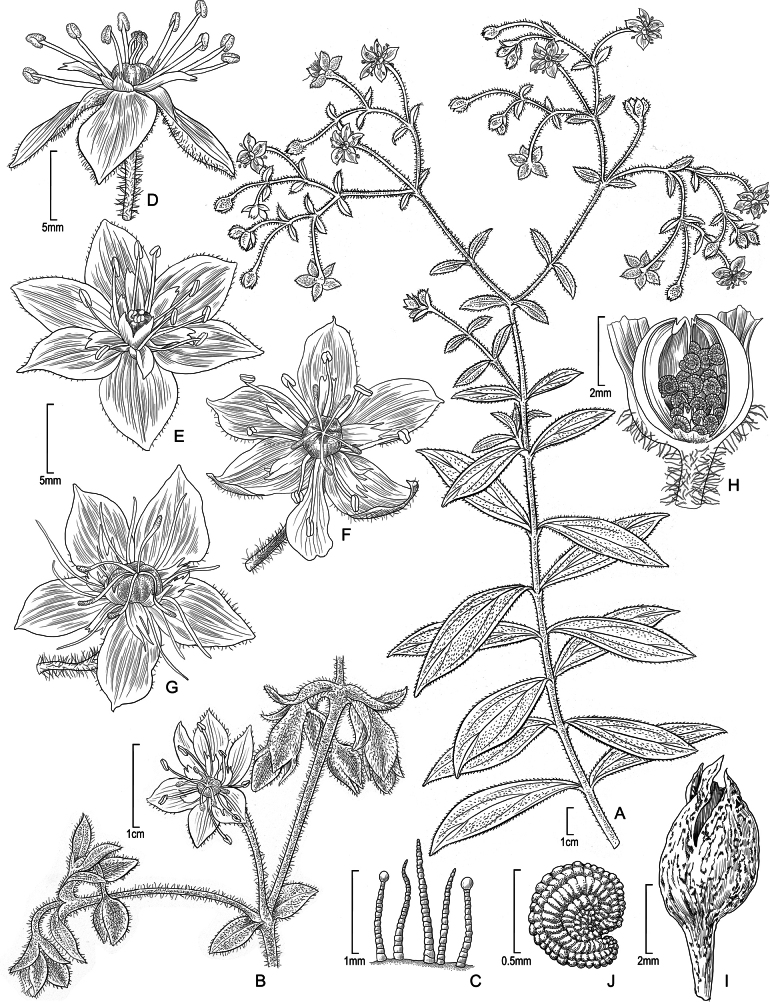
*Schiedeawaiahuluensis***A** habit, stem with leaves and inflorescence **B** branch of inflorescence, showing open flower with 6 sepals **C** hair types from petiole **D** flower, lateral view showing anthers at male stage **E** flower with 6 sepals and showing rarely dissected nectar shafts, face view **F** flower with 5 sepals, single petal, face view **G** flower with 5 sepals, female stage **H** fruit cut open to reveal seeds **I** dry fruit **J** seed. Drawn from living material from cultivated individual at University of California, Irvine (UCI) greenhouse (**A, C, F, G, H, J**), from photographs of cultivated material at UCI (**B, D, I**) and photograph taken by drone (**E**). Illustration by Alice Tangerini.

**Figure 2.**
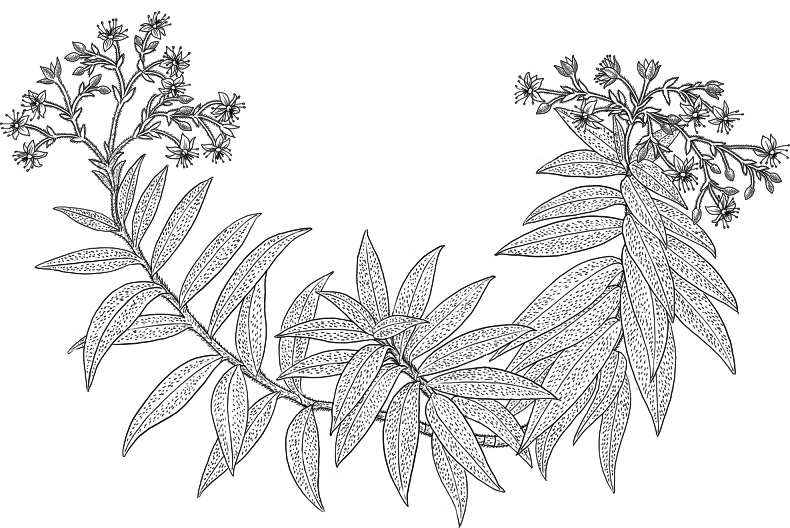
*Schiedeawaiahuluensis* showing habit with stem, leaves, and inflorescence growing in wild on edge of rock wall. Drawn from photographs taken by drone in wild and of collected stems (20220607_PuuKaPele-3, IMG_5929, IMG_5939). Illustration by Alice Tangerini.

**Figure 3.**
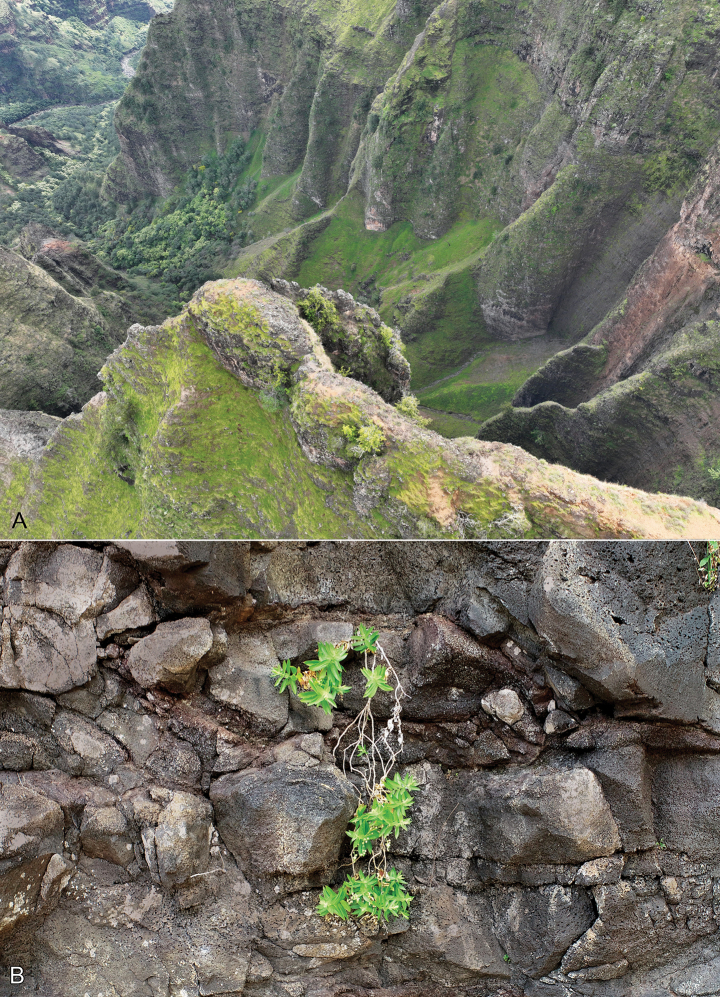
*Schiedeawaiahuluensis* habitat **A** Waiahulu branch of Waimea Canyon, drone photo, by Ben Nyberg **B** non-collected individual, drone photo by Ben Nyberg.

**Figure 4.**
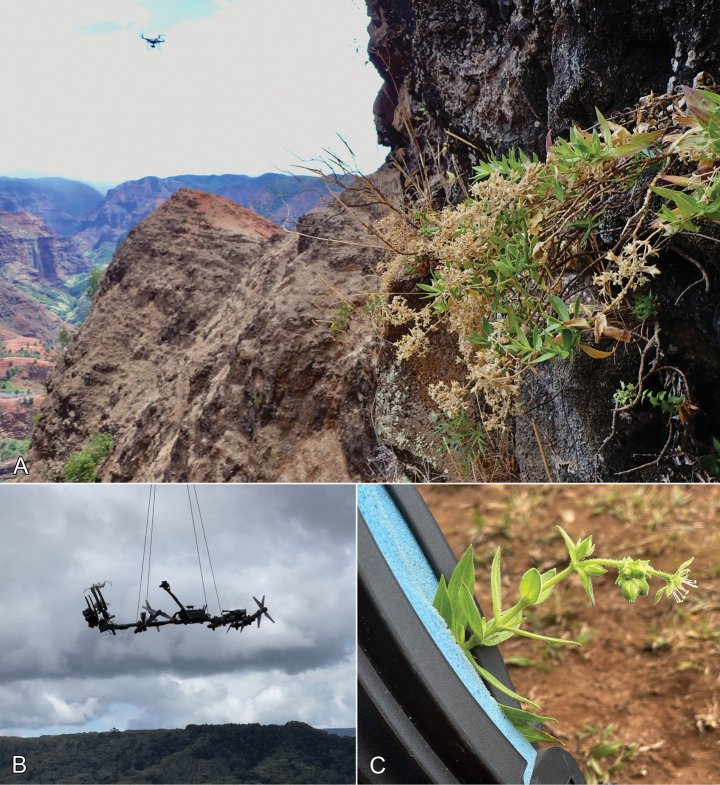
Collecting *Schiedeawaiahuluensis* via drone **A** population accessed on rope, with drone in background *William AMW821*, photo by Adam Williams **B** collecting arm hanging from drone, photo by Ben Nyberg **C** drone collecting arm with specimen, *Nyberg BN023* photo by Ben Nyberg.

**Figure 5.**
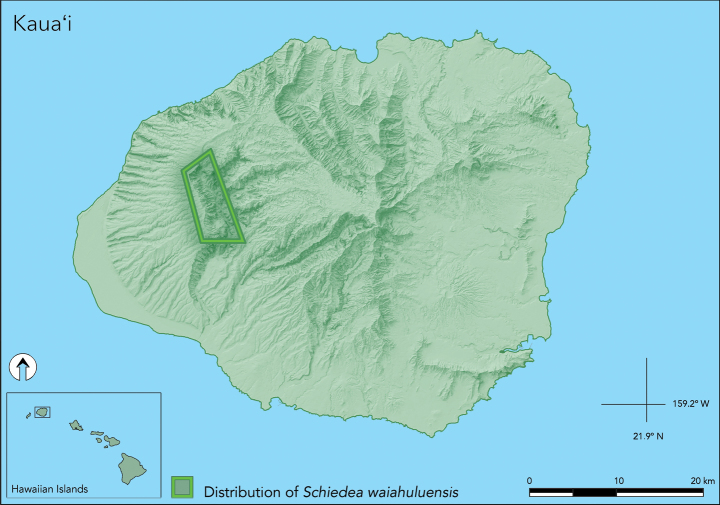
Map of Kaua'i, Hawaiian Islands, with rectangle indicating general distribution of *Schiedeawaiahuluensis*.

**Figure 6.**
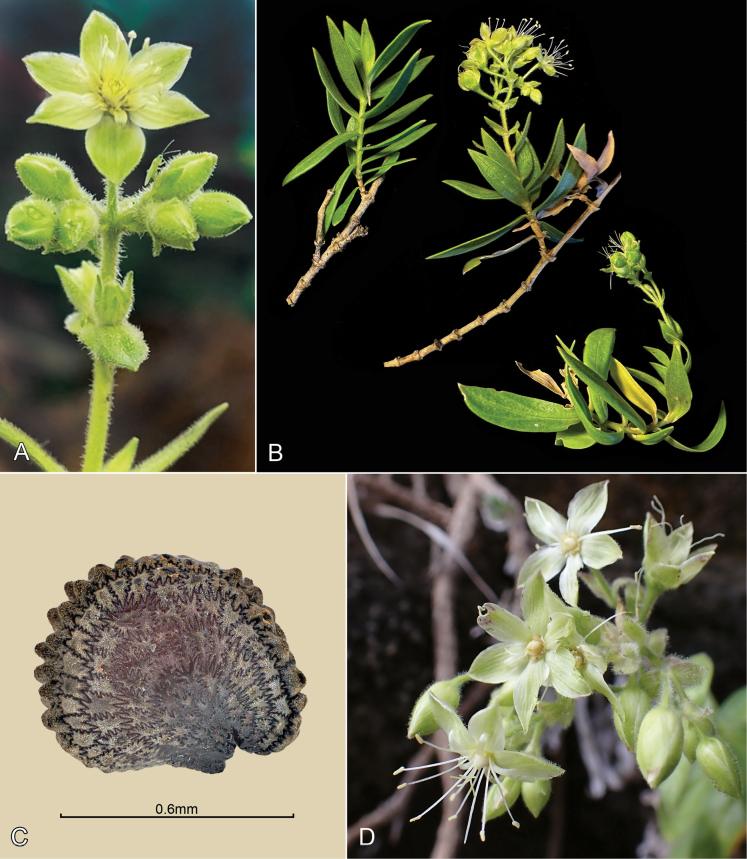
*Schiedeawaiahuluensis***A** drone collected specimen *Nyberg BN 023* with endemic Mirid on upper right bud **B** habit of plant in native habitat *Williams AMW820*, photo by Ben Nyberg **C** seed *William AMW 821*, photo by seedsofhawaii.org**D** flower of drone collected specimen, *Nyberg BN 023*, photo by KR Wood.

Figs 1 , 2 , 3 , 4 , 5 , 6

#### Type.

**USA** • **Hawaiian Islands, Kaua'i**: Cultivated at University of California, Irvine from seed collected at Waimea District, north-facing dry cliffs above Waiahulu, 767 m alt., 29 March 2022, *B. Nyberg et al. BN023* (PTBG), 12 April 2024, *S. G. Weller & A. K. Sakai 1172* (holotype: PTBG 1000097349!; isotypes: BISH!, G!, K!, MO!, UC1, US!).

#### Description.

Erect to spreading shrub to 40 cm long; stems terete, green, glabrous in young plants, but becoming conspicuously viscid, glandular pubescent throughout as plants age, ascending, sprawling, becoming pendent as they elongate in the wild, much branched, with side branches elongating to length of main axis and flowering at the same time as main axis. ***Leaves*** opposite, 4.5–7 (–11.9 in cultivation) cm long, 1.5–2.1 (–3.3 in cultivation) cm wide, thin, green, narrowly oblanceolate, mid-rib prominent and 2–5 conspicuous veins branching from the mid-vein near the base of the leaf, glandular pubescent on mature plants, margin entire, slightly thickened, apex acute, base gradually tapering, sessile. ***Inflorescence*** terminal, erect, with (4–)9–27(–45) flowers, pseudo-axillary or with a main axis 20.5–36.5 cm long; bracts green, densely glandular pubescent, the lowermost 13–29 mm long, 7–13 mm wide, those of the distal branches and flowers 5–12 mm long, 3–8 mm wide; pedicels spreading at anthesis, densely glandular pubescent (3–) 5–30 (–40) mm long at anthesis. ***Flowers*** hermaphroditic, strongly protandrous. Sepals (4–) 5–6, usually subequal in size, 4.5–11.9 mm in length and 2.5–5 mm in width, occasionally two narrower sepals positioned on opposite sides of the flower, green or whitish-green on adaxial face, occasionally with distinctive whitish margins, concave, ovate, orientated at ca. 90° angle to pedicel, adaxial side glabrous, abaxial side glandular pubescent, margins entire, scarious, apex apiculate. Nectary shaft tubular and notched at apex, rarely dissected in up to 3 parts, flap-like to tubular or partially tubular, 2.0–5.0 mm in length, recurved and appressed at the tip to the opposed sepal, small quantities of pale brown or rarely black nectar produced and, in the greenhouse, released on to subtending sepal as stigmas become receptive. Stamens 8–10(–12), 5.3–10 mm long; anthers ca. 0.9 mm long, yellow, dehiscing after flower opens, but before stigmas become receptive. Styles 5–8(–11), 3.6–10.0 mm in length when receptive, stigmatic papillae on distal half of style. ***Ovary*** 1.5–3.0 mm in width, 1.0–2.0 mm in height. ***Capsules*** 2.6– 3.2 mm long, subglobose, apparently tardily dehiscent. ***Seeds*** 13–82, viable following self-pollinations in greenhouse, black, 0.4–0.6 (–0.7) mm long, orbicular-reniform, compressed, the faces transversely rugose, the margins papillose. Chromosome number unknown.

#### Etymology.

Specific epithet refers to the Waiahulu cliff region of Waimea Canyon, Kaua'i, the only known location where the new species is found.

#### Specimens examined.

**United States. Hawaiian Islands, Kaua'i**: Waimea District, north-facing dry cliffs above Waiahulu, 767 m alt., 29 Mar 2022, *B. Nyberg et al. BN023* (PTBG) • Ridge below the Waimea Canyon Lookout, Waiahulu region of Waimea Canyon Complex, Pu'u Ka Pele Forest Reserve, 809 m alt., 12 Sep 2023, *Williams et al. AMW818–AMW825* (PTBG), *Wood et al. 19397* (PTBG) • NTBG Horticultural Center, 12 Jul 2024, *K.R. Wood et al. 19557* (PTBG).

#### Distribution, habitat and threats.

*Schiedeawaiahuluensis* is endemic to the Hawaiian Islands where it is narrowly restricted to the western side of Kaua'i and occurs in open dry to mesic cliff habitat (1000–1500 mm rain/year) above Waiahulu Stream, which occurs in a small tributary of the greater Waimea Canyon located in the Pu'u Ka Pele Forest Reserve (Fig. 5). To date we have now established the distribution of *S.waiahuluensis* to extend ca. 5 km north to south along the basalt cliffs above Waiahulu Stream, with an estimated population of ca. 345 individuals ranging between 530–950 m elev. The majority of the plants are growing on vertical bare rock in minute cracks with small pockets of soil. Occasional observations include plants growing on rock shelves or under overhanging cliffs.

Although the native vegetation in Waimea Canyon has been seriously degraded by goats (*Caprahircus* L.) since their introduction in the late 1790s, there are still rich patches of endemic plant species found in the more inaccessible sections of the forest reserve, especially the vertical dry cliffs. Relictual native shrubs and trees associated in the region where *S.waiahuluensis* occurs include *Nototrichiumsandwicense* (A.Gray) Hillebr. (Amaranthaceae); *Peucedanumsandwicense* Hillebr. (Apiaceae); *Artemisiaaustralis* Less., BidenssandvicensisLess.ssp.confusa Nagata & Ganders, *Wollastoniafauriei* (H.Lév.) Orchard, *Wilkesiagymnoxiphium* A.Gray (Asteraceae); *Lobelianiihauensis* H.St.John (Campanulaceae); EuphorbiacelastroidesBoiss.var.hanapepensis Sherff (Euphorbiaceae); *Argemoneglauca* (Nutt. ex Prain) Pope (Papaveraceae); *Dodonaeaviscosa* Jacq. (Sapindaceae); and *Neraudiamelastomifolia* Gaudich. (Urticaceae). Two native grasses commonly found in this habitat include *Eragrostisvariabilis* (Gaudich.) Steud. and *Panicumlineale* H.St.John (Poaceae), along with the fern *Doryopterisdecora* Brack. (Pteridaceae). *Isodendrionpyrifolium* A.Gray (Violaceae), a small federally endangered shrub previously unrecorded from Kaua'i, was also discovered growing with *S.waiahuluensis* (Nyberg et al. 2023) along with *S.apokremnos* H.St.John and *S.spergulina* A.Gray (Caryophyllaceae).

Although at least a portion of the sheer vertical cliff habitat of *Schiedeawaiahuluensis* has apparently escaped degradation by feral goats, the former distribution of this species may have been more extensive prior to the introduction of goats. In addition to their immediate negative effects on native plant species, goats in this region aid in the dispersal of invasive non-native plant species, most notably *Plucheacarolinensis* (Jacq.) G.Don (Asteraceae); *Hyptispectinata* (L.) Poit. (Lamiaceae); *Festucabromoides* L. (Poaceae), *Pentapogonmicranthus* (Cav.) P.M.Peterson, Romasch. & Soreng (Poaceae); *Grevillearobusta* A.Cunn. ex R.Br. (Proteaceae); and *Lantanacamara* L. (Verbenaceae).

#### Preliminary conservation assessment.

Critically Endangered - B1ab(i,ii,iii,v)

*Schiedeawaiahuluensis* was assessed for endangerment using the IUCN criteria. Its limited geographic range in Extent of Occurrence (EOO, 2.5 km^2^) along with its single location and inferred decline of habitat quality led to its classification as Critically Endangered (CR). Further surveys of adjacent cliff habitats are necessary to better understand the distribution and abundance of *S.waiahuluensis*. Establishment and replication of ex-situ collections is underway; additional collections from unrepresented sub-populations would add to better understanding of the genetic diversity of this species.

## ﻿Discussion

The distinctive morphology of this remarkable new species places *Schiedeawaiahuluensis* into sect. Leucocalyx W.L.Wagner & Weller, previously represented by a single cliff-dwelling species, *S.attenuata* W.L.Wagner, Weller & A.K.Sakai. Besides both being cliff-dwellers with a woody habit, they are also both hermaphroditic, with enlarged greenish-white sepals, coloured nectar and 5 to 7 or 8 styles. *Schiedeaattenuata* occurs in Kalalau Valley, around 7 km to the north of *S.waiahuluensis* and is restricted to dry/mesic vertical cliffs. Morphological characters of these species and *S.viscosa* are compared in Table 1. The enlarged, somewhat whitish sepals of *S.waiahuluensis* (Fig. 6), glandular pubescence and coloured nectar are features also found in *S.viscosa* H.Mann and *S.lychnoides* Hillebr. (sect. Nothoschiedea H.Mann). Ongoing analyses (McDonnell et al., in prep.) of our study of the phylogenetics of the genus *Schiedea* using Hyb-Seq showed that addition of a single sample of *S.waiahuluensis* placed it as sister to *S.attenuata*. This clade was, in turn, sister to a clade consisting of sect. Nothoschiedea + sect. Alsinidendron. Additional samples of *S.waiahuluensis* that will be added to the analyses should help with further understanding of the phylogenetic placement of *Schiedeawaiahuluensis* and other details of the diversification of the genus.

**Table 1.** Comparison of morphological characters of the subclade of species of Schiedeasect.Leucocalyx and *S.viscosa* (sect. Nothoschiedea).

Character	*S.waiahuluensis*	*S.attenuata*	*S.viscosa*
Stem shape	Terete	Compressed-terete	Terete
Pubescence	Glabrous in young plant, becoming densely viscid, glandular pubescent	Glabrous to very sparsely puberulent with minute hairs	Moderately viscid glandular puberulent
Leaf length and width (cm)	4.5–7(–11.9) × 1.5–2.1 (–3.3) cm	5.3–7(–12) × 0.5–0.7(–1.1) cm	2.5–5 × 0.8–1.8 cm
Sepal number and aspect	(4–)5–6, usually subequal in size, slightly concave	5, equal, navicular towards apex	4–5, flat
Sepal length	6–12 mm	4–5 mm	6.5–12 mm
Nectary	Tubular and notched at apex, rarely dissected in up to 3 parts, flap-like to tubular or partially tubular	Tubular and notched at apex	Flap-like
Nectar colour	Brown or occasionally black	Clear	Black
Style number	5–8(–11)	5–6(–7)	5–7(–8)
Capsule length	2.6–3.2 mm	3.3–4.5 mm	8–12 mm

In contrast to the facultatively self-pollinating species in sect. Nothoschiedea, *S.waiahuluensis* appears to be outcrossing, based on strong protandry and production of ca. 16,000 pollen grains per flower, a value typical of outcrossing species (Weller et al. 1998). The large, spreading sepals of this species suggest biotic pollination, although field studies to test this possibility would be extremely challenging. *Schiedeawaiahuluensis* is unique amongst *Schiedea* species for the discovery of a native, probably new species of *Engytatus* (Heteroptera, Miridae; Polhemus, pers. comm.) on several individuals surveyed on the cliffs of Waiahulu Canyon (Fig. 6A). These are the first observations of a potential native herbivore on a *Schiedea* species.

Increasingly, drones are being used to assess and inventory cliff-dwelling plant taxa (Strumia et al. 2020; Reckling et al. 2021; Gao et al. 2024). The addition of drone-based sampling tools has the potential to transform plant conservation efforts in these hard-to-reach cliff environments (La Vigne et al. 2022). Hidden floristic diversity is likely to emerge as we embark on this new era of exploration and documentation of cliff ecosystems. The discovery of *S.waiahuluensis* after over 40 years of intense interest in this genus on Kaua'i indicates the potential for new discoveries using drone technology in studies of other endemic plant genera in the Hawaiian Islands.

## Supplementary Material

XML Treatment for Schiedea waiahuluensis

## ﻿Additional information

### Author ORCIDs

Warren L. Wagner https://orcid.org/0000-0001-5012-8422

Ben Nyberg https://orcid.org/0000-0002-5894-5088

Kenneth R. Wood https://orcid.org/0000-0001-6446-1154

### Data availability

All of the data that support the findings of this study are available in the main text.
